# Factors affecting timing of closure and non-reversal of temporary ileostomies

**DOI:** 10.1007/s00384-015-2253-3

**Published:** 2015-06-09

**Authors:** M. F. Sier, L. van Gelder, D. T. Ubbink, W. A. Bemelman, R. J. Oostenbroek

**Affiliations:** Department of Surgery, Albert Schweitzer Hospital, Location Dordwijk, Room: W0-051, Albert Schweitzerplaats 25, 3318 AT Dordrecht, The Netherlands; Department of Surgery, Academic Medical Center, University of Amsterdam, Amsterdam, The Netherlands

**Keywords:** Defunctioning stoma, (temporary) Ileostomy, Reversal, Takedown, Prediction

## Abstract

**Background:**

Although stoma closure is considered a simple surgical intervention, the interval between construction and reversal is often prolonged, and some ileostomies may never be reversed. We evaluated possible predictors for non-reversal and prolonged interval between construction and reversal.

**Material and methods:**

In a cohort study of ileostomy patients treated in a large teaching hospital, we collected data from the surgical complication and enterostomal therapists’ registries between January 2001 and December 2011. Parameters responsible for morbidity, mortality, length of stay and time interval between construction and reversal were analysed.

**Results:**

Of 485 intentionally temporary ileostomies, 359 were reversed after a median of 5.6 months (IQR 3.8–8.9 months), while 126 (26 %) remained permanent.

End ileostomy and intra-abdominal abscess independently delayed reversal. Age, end ileostomy, higher body mass index and preoperative radiotherapy were independent factors for non-reversal. Median duration of hospitalisation for reversal was 7.0 days (5–13 days). Morbidity and mortality were 31 and 0.9 %, respectively. In 20 patients (5.5 %), re-ileostomy was necessary.

**Conclusions:**

A substantial number of ileostomies that are intended to be temporary will never be reversed. If reversed, the interval between construction and reversal is longer than anticipated, while morbidity after reversal and duration of hospitalisation are considerable. Besides a temporary ileostomy, there are two other options: no diversion or a permanent colostomy. Shared decision-making is to be preferred in these situations.

## Introduction

Although the potential benefits of a defunctioning ileostomy are clear, stoma-related morbidity is known to be high [1, 2]. Recent literature shows overall stoma-related morbidity among various patient populations, and using different types of stomata may vary between 17 and 45 % [1–3]. Stomata have also been shown to negatively influence quality of life and body image [4, 5]. Despite prior counselling, many patients remain distressed with the thought of having a stoma and are keen to get it closed as early as possible. Hence, both surgeons and patients look forward to an early closure of the stoma.

When creating a diverting ileostomy, the aim is to reverse it after 6–12 weeks [2, 6, 7].

There are no set protocols for stoma closure [8]. Scheduling of reversal is extremely variable among hospitals [9]. In several studies, time to closure was considerably longer (ranging from 13 to 37 weeks). This may be due not only to prolonged recovery following initial surgery, development of complications after creation and adjuvant treatment but also administrative delays such as waiting lists for cancer surgery or other urgent procedures [2, 10]. As a result, patients sometimes retain their stoma much longer than initially proposed, with an inherent impact on their physical and psychological well-being as well as on the healthcare budget [10]. Furthermore, not all temporary ileostomies are reversed; 3–25 % of these ileostomies become permanent [1, 9–11].

Even though ileostomy reversal is generally considered a simple procedure, it can also have a severe impact on the patient, with morbidity rates up to 45 % [2, 12, 13]. A complicated clinical course after closure can also have a deleterious effect on the patient’s quality of life.

The aim of this study was to assess various aspects of stoma closure, i.e. frequency of closure, time interval, morbidity (e.g. anastomotic leakage, enterocutaneous fistula, postoperative bleeding, stoma site infection), mortality, possible risk factors for delay in reversal and possible predictors for non-closure in an 11-year cohort of ileostomy patients to better counsel patients (and surgeons) on a possibly delayed or even non-reversal of an ileostomy.

## Patients and methods

The conduct and description of this cohort study were performed according to the STROBE statement [14].

### Design and setting

In this patient cohort, all ileostomies constructed between January 2001 and January 2012 in the Albert Schweitzer Hospital in Dordrecht, a large secondary referral and teaching hospital in the Netherlands, were studied. Patient follow-up was until March 2013.

### Patient selection

We searched for all patients (children and adults) who had undergone a loop or end ileostomy and selected those who were intended to be reversed. Excluded were patients who had received an ileostomy deemed permanent from the beginning.

Eligible patients were retrieved from the database of the enterostomal therapists, who were involved in the instruction, preparation and follow-up care of all patients receiving a stoma. We also searched the hospital’s surgery registration database for stoma reversal procedures to make sure all patients from that period were included.

### Reversal procedure

A colorectal surgeon or a surgical resident under the direct supervision of a colorectal surgeon carried out closure of the ileostomy under general anaesthesia. All patients were given antibiotic prophylaxis: amoxicillin and clavulanic acid 2.2 g and aminoglycoside 4 mg per kg intravenously 1 h preoperatively. Ileostomies were closed by means of a running suture or a stapled closure, depending on the preference of the (supervising) surgeon.

The stoma was dissected from the mucocutaneous junction and delivered from the rectus sheath and peritoneal cavity by sharp dissection. Loop ileostomies were closed extraperitoneally (if possible) and end ileostomies intraperitoneally by means of a running suture or a stapled closure, depending on the preference of the (supervising) surgeon. The posterior and anterior rectus sheaths were closed to minimise the risk of an incisional hernia. The wound was partially left open to prevent wound infections. After surgery, the nasal gastric tube was removed, and the patient started a normal diet as soon as possible. Patients were discharged if they tolerated a normal diet, had stool and could take care of the wound. The patient was scheduled to visit the outpatient clinic 2 weeks after discharge.

### Data collection

Patient characteristics, surgery-related data and stoma-related complications were collected from the hospital’s surgical complication registration Database combined with the prospectively collected dataset of the enterostomal therapists (EI version 4.0 for Windows, Combicare, Gouda, the Netherlands). Two surgical residents collected the data. A standard data extraction form was used in the retrieval process. Data were checked for completeness and accuracy by means of random sampling by one of the supervising surgeons. A complication was defined as a postoperative, stoma-related complication.

We registered age, gender, body mass index (BMI), American Society of Anesthesiologists (ASA) classification, indications for ostomy surgery (malignancy, inflammatory bowel disease, diverticulitis, complications after surgery, ileus or trauma), comorbidities, time between stoma construction and reversal, morbidity, mortality rate and reoperations after reversal, when and why a new stoma had to be constructed, as well as follow-up data of the patients. “Non-reversal” was defined as a stoma being still present at the end of follow-up. Reasons for not closing the stoma were recorded and analysed.

### Statistical analysis

Outcome variables are presented as means and standard deviations (SD) or medians and interquartile ranges (IQR), if not normally distributed. Differences in dichotomous outcomes are expressed as risk differences including their 95 % confidence intervals (CI). Differences in continuous variables were analysed with the Mann-Whitney *U* test because of the non-normal distribution. Kaplan-Meier curves including log-rank testing were used to present and compare crude proportions of non-reversal.

Multivariable stepwise logistic regression analysis was performed to detect independent factors associated with eventual stoma closure, expressed as odds ratios (OR) with 95 % CIs. Based on this multivariable logistic regression analysis, we defined a formula to predict the chance of non-reversal.

Also a multivariable Cox proportional hazards analysis was done to calculate the hazard ratios (HR) of factors influencing the time interval before stoma closure. Differences were considered significant at *P* < 0.05. Factors possibly predicting non-reversal or delayed closure of the ileostomy were selected based on clinical relevance and prior univariable analysis.

All calculations were performed using the Statistical Package for the Social Science (SPSS) for Windows (version 20; IBM SPSS Inc., Armonk, NY, USA).

## Results

### Participants

**Fig. 1:**
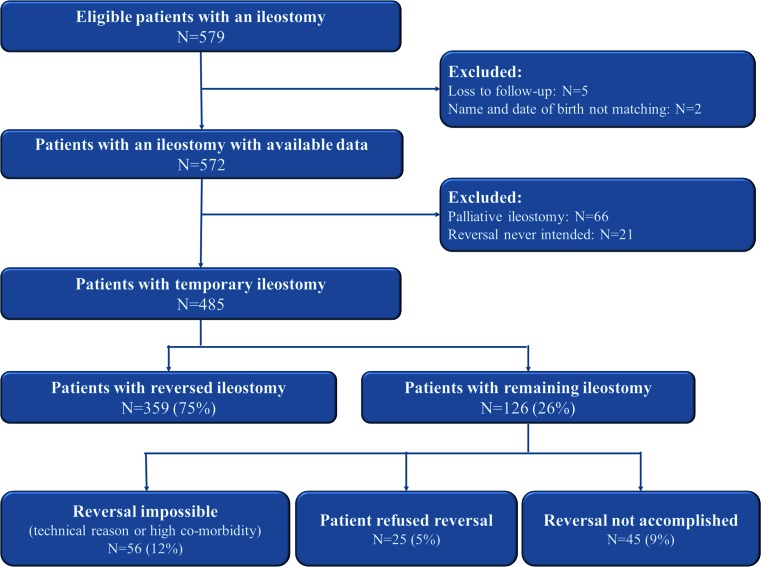
Overview of patient inclusions and outcomes

Between 2001 and 2012, 572 ileostomies were constructed. Of these, 485 were intended to be temporary. The flow chart of patient inclusion is shown in Fig. 1.

Baseline characteristics of the included patients are displayed in Table 1. Most of the patients in this study received a loop ileostomy to divert low anastomoses after rectal excision for malignancy. An end ileostomy was performed mostly in patients who needed a resection for irritable bowel syndrome, for example, a proctocolectomy or a subtotal colectomy. Median follow-up was 22.6 months (IQR 10.1–46.0).

**Table 1:** Baseline patient characteristics

Gender	Male	207 (58 %)	
Age (years)		64.3	IQR (54.7–72.8)
BMI		25	IQR (23.0–28.8)
ASA	1	73 (20 %)	
	2	228 (64 %)	
	≥3	58 (16 %)	
Ileostomy	Loop	289 (81 %)	
	End	70 (19 %)	
Surgery	Elective	304 (86 %)	
	Acute	50 (14 %)	
Reason	Malignancy	217 (60 %)	
	Benign	142 (40 %)	

Data are presented as percentages or medians with interquartile ranges (IQR)

*BMI* body mass index, *ASA* American Society of Anaesthesiologists classification

The stoma remained permanent in 126 of the 485 patients (26 %). In 56 patients, closure was considered contraindicated because of high comorbidity (9.8 %). Reversal was refused by 25 patients (4.4 %), one patient had its ileostomy closed in another hospital, and in 45 patients, reversal was not accomplished (7.9 %) of whom 21 patients died after initial surgery, for example, because of anastomotic leakage or bowel ischemia. Twelve patients died because of other reasons, like exacerbation of ulcerative colitis, non-Hodgkin lymphoma, kidney failure, myocardial infarction or disease progression. At the last follow-up, 7 patients still had active disease and reversal was not yet possible, for example, because of perianal fistula, presacral sinus and disease recurrence. In 5 patients, it was not clear why reversal had not yet been accomplished.

In 20 patients, the stoma had to be reconstructed again, for example, due to anastomotic leakage, stenosis and incontinence; 11 patients after reversal of a loop ileostomy and 9 patients after reversal of an end ileostomy. We found a risk difference of 9.2 % (0.1 %–17.4 %) in favour of the loop ileostomy group.

### Ileostomy reversal

Stoma reversal rates for each of the underlying diseases are shown in Table 2. Stoma reversal was possible in 79 % after surgery for malignancy, in 67 % for benign disease and in 82 % of the loop ileostomies and 53 % of the end ileostomies; 26 %(*n* = 126) of the intentionally temporary ileostomies were never reversed, while in patients with stomata that were intended to be closed, 88 % of these were closed within a year (Table 2).

**Table 2:** Reversal rates per indication

Indication	Reversed ileostomies	Total ileostomies
Malignancy	210 (79 %)	267
Inflammatory bowel disease	30 (59 %)	52
Diverticulitis	51 (80 %)	64
Ileus	10 (63 %)	16
Complication after surgery	35 (85 %)	41
Other reasons	23 (51 %)	45
Total	359 (74 %)	485

### Risk factors for non-reversal

In the multivariable logistic regression analysis, the following possibly associated factors were entered: construction of the ileostomy in an elective or in an acute setting, stoma-related complications after stoma creation as well as anastomotic leakages despite defunctioning stoma (for example leading to a presacral sinus), stoma retraction and so on, the responsible surgeon, type of ileostomy, (neo) adjuvant therapy, ASA classification, BMI, re-laparotomy after creation, revision of stoma and indication for surgery. These factors were selected based on clinical relevance and the univariable analysis.

Independent predictors for non-reversal were found to be end ileostomy, preoperative radiotherapy, BMI and higher age (Table 3). The odds for reversal in patients with a loop ileostomy were 4.3 times higher than in patients with an end ileostomy. The odds for reversal decreased with 3 % per year increase in age (OR 0.97). Each point increase in BMI was associated with a small but significant 7 % higher chance of reversal. Anastomotic leakage was not found to be an independent predictor of eventual non-reversal.

**Table 3:** Results of multivariable logistic regression analysis

	*B*	*P* value	OR	95 % CI for HR	
				Lower	Upper
Age	−0.034	0.000	0.967	0.949	0.985
Preoperative radiotherapy	−1.030	0.007	0.357	0.170	0.751
Loop ileostomy	1.454	0.000	4.280	2.288	8.007
BMI	0.065	0.028	1.067	1.007	1.130
Constant	1.535	0.094	4.639		

OR < 1 indicates decreased likelihood for stoma reversal

Based on our multivariable logistic regression analysis, we could define the following formula to predict the chance of reversal when a patient has one or more of the risk factors found (Table 3).

$$ \begin{array}{c}\hfill \mathrm{L}\mathrm{n}\ \left(\mathrm{odds}\ \mathrm{f}\mathrm{o}\mathrm{r}\ \mathrm{n}\mathrm{o}\mathrm{n}\hbox{-} \mathrm{reversal}\right) = 1.535-0.34 \times \mathrm{age}\hfill \\ {}\hfill - 1.030 \times \mathrm{preoperative}\ \mathrm{r}\mathrm{adiotherapy}\hfill \\ {}\hfill + 1.454 \times \mathrm{loop}\ \mathrm{ileostomy}\hfill \\ {}\hfill + 0.65 \times \mathrm{B}\mathrm{M}\mathrm{I}\hfill \end{array} $$

For example, if we have a patient of 50 years old with a BMI of 24 and who underwent preoperative radiotherapy and received a loop ileostomy, the chance that the ileostomy will not be closed is 64 %:

$$ \begin{array}{c}\hfill P\left(\mathrm{n}\mathrm{o}\mathrm{n}\hbox{-} \mathrm{reversal}\right)=1/\left(1+{\mathrm{e}}^{-}{{}^{\Big(1}}^{\times }{{}^{535-0}}^{\times }{{}^{34\ \mathrm{x}50 - 1}}^{\times }{{}^{030\ \mathrm{x}\ 1 + 1}}^{\times }{{{}^{454\ \mathrm{x}\ 1 + 0}}^{\times}}^{65\ \mathrm{x}\ 24\Big)}\right)\hfill \\ {}\hfill P\ \left(\mathrm{n}\mathrm{o}\mathrm{n}\hbox{-} \mathrm{reversal}\right)=1/\left(1+{\mathrm{e}}^{- \Big(0}{{}^{\times}}^{559\Big)}\right)=0.636\hfill \end{array} $$

### Risk factors for delay in reversal

Median time to reversal was 5.6 months (IQR 3.9–9.0). In the multivariate Cox regression analysis, the following factors were entered: construction of an ileostomy in an elective or in an acute setting, (stoma-related) complications after creation, operating surgeon, type of ileostomy, (neo) adjuvant therapy, ASA classification, BMI, re-laparotomy after creation, revision of stoma and indication for surgery. These factors were selected based on clinical relevance and the univariable analysis.

**Fig. 2:**
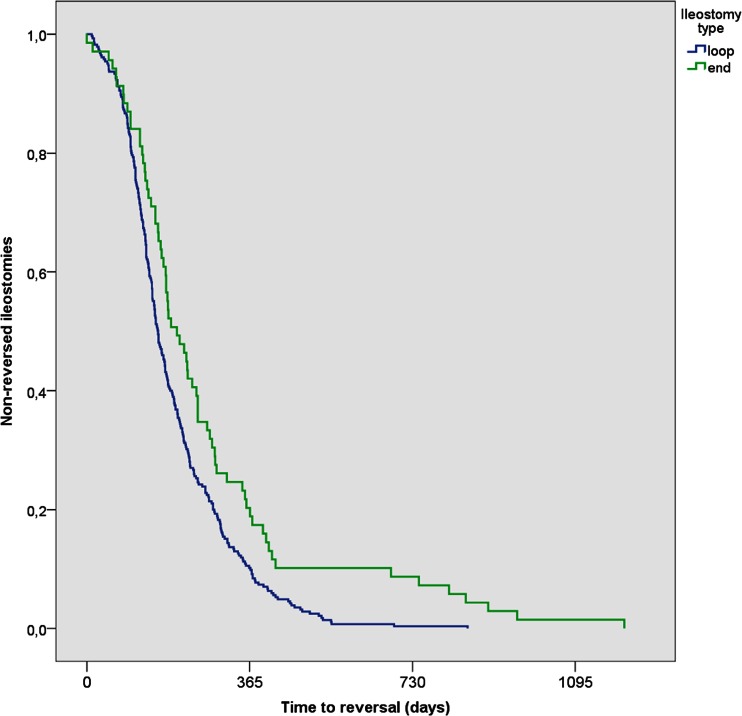
Kaplan-Meier curve of the number of not (yet) reversed

Independent factors for delayed reversal were an end ileostomy, an intra-abdominal abscess, diverticulitis or complications after initial surgery necessitating the construction of an ileostomy (Table 4). As shown in Table 4 and Fig. 2, when considering the eventually reversed ileostomies, loop ileostomies were reversed significantly faster than end ileostomies (HR 0.378, *P* < 0.001). Patients with an intra-abdominal abscess after initial surgery had a higher risk of delay in reversal than patients without an abscess (HR 0.706, *P* = 0.021). Ileostomies in patients with diverticulitis and in patients who had a complication after initial surgery were reversed significantly sooner than for a malignancy (resp. HR 1.424, *P* = 0.026 and HR 1.827, *P* = 0.002).

**Table 4:** Results of multivariate Cox regression analysis

	*P* value	HR	95 % CI for HR	
			Lower	Upper
End ileostomy	0.000	0.378	0.275	0.520
Abscess	0.021	0.706	0.526	0.948
Reason for surgery compared to malignancy	0.009			
Diverticulitis	0.026	1.424	1.044	1.942
Complication after initial operation	0.002	1.827	1.250	2.670

HR < 1 indicates higher risk of delayed reversal

Median hospital stay was significantly (*P* < 0.001) longer after closure of end ileostomies (11 days, IQR 7.50–17.50) as compared with loop ileostomies (7 days, IQR 5.0–11.5). No trend towards a shorter hospital stay over the years was observed.

Postoperative complications occurred in 112 of the 359 reversal procedures (31 %), while 20 patients sustained more than one complication (5.5 %). In 20 patients, a new stoma had to be constructed because of complications (6 %); in 4 % of the loop ileostomies that were closed and 12 % of the end ileostomies. Reasons for the creation of a new stoma were anastomotic leakage, stenosis, abscesses, fistula or pouchitis. Stoma-related mortality after stoma reversal was 0.9 % (*n* = 5).

## Discussion

Although literature suggests that temporary ileostomies can safely be reversed in due time, the interval between construction and reversal is often very long, and a large proportion of the stomas will never be reversed. The present study shows that in a large cohort of temporary stomas, only 71 % of the loop ileostomies and 43 % of the end ileostomies were closed eventually. Median interval to closure was nearly half a year.

The non-reversal percentage in this study is even higher than previously observed in the literature. Other studies showed non-reversal rates ranging from 9 to 25 % [1, 2, 9, 12, 15]. This discrepancy can in part be explained by our study population, which also included patients who received an ileostomy for other reasons than a malignancy.

Older age, lower BMI, end ileostomy and preoperative radiotherapy are found to be independent factors associated with non-reversal. Besides, our prediction model indicates that if a patient would have all risk factors, he has a 64 % risk that his ileostomy will not be reversed. The literature confirms our finding that age is associated with non-reversal [1, 9, 11, 16]. In patients over 70 years, one out of three ileostomies became permanent [9]. Probably this is due to a higher comorbidity rate and unwillingness to be operated again. Den Dulk et al. found similar results for end ileostomies [1]. However, they also found postoperative complications and recurrence to be risk factors, which we could not confirm.

End ileostomies rather than loop ileostomies are more prone to a delay before closure and even to become permanent. Loop ileostomies are typically constructed to divert a downstream anastomosis and can be closed locally. End ileostomies are more often made after intestinal resection where immediate re-anastomosing is considered to be unsafe. Perhaps this also explains why after closure of an end ileostomy, the chance of reconstruction of a new stoma due to complications after reversal is higher due to the fact that closure must be done by laparotomy or laparoscopy. Surgeons should be aware of these disappointing figures and include these in their decision-making about creating a temporary stoma against the risk of increased morbidity or deciding for a permanent stoma. We believe that these disappointing results are due to the fact that especially during the first years of the study, almost all surgeons in our hospital and probably also in our country were quite conservative in performing a primary anastomosis protected by a loop ileostomy and preferred an end ileostomy without an anastomosis.

In our study, preoperative radiotherapy reduced reversal rates. However, in the study of Lindgren et al., preoperative radiotherapy was not an independent risk factor for non-reversal [11]. Furthermore, in our experience, a small group of patients eventually accepted having a stoma for a longer period, as they preferred to be disease-free after their eventful period during the past year.

Diverting ileostomies are mostly constructed to protect a downstream anastomosis. It is known that the leakage rate will be reduced by one third to one half and that the clinical consequences are mitigated [7, 17, 18]. Depending on the leak rate of the specific anastomosis, most diverting ileostomies are constructed only as a precaution. A balanced decision should be made with the patient whether or not to protect the downstream anastomosis. Since the non-closure rate of end ileostomies is considerable, one could argue that re-anastomosing combined with a diverting ileostomy might be the better option or even to choose for no diversion or a permanent colostomy. These considerations make shared decision-making with the patient important [19]. The same accounts for the decision to restore the continuity after rectal resection.

Existing literature does not provide a protocol for optimal closure and timing of closure for ileostomies [8]. However, most surgeons would prefer to close the ileostomy as soon as the patient is medically fit and willing [10]. After 10–14 days, inflammatory adhesions around the stoma will interfere with stoma closure. A period of at least 6–10 weeks is required for the inflammatory adhesions to subside. It has been shown that in selective patients, e.g. those who recover quickly after the initial surgery, the stoma can be closed within these 10–14 days avoiding a longer period of having a stoma with the associated problems and costs [6, 17]. The majority of patients experience an overall improvement of quality of life, physical functioning and social functioning following stoma closure [20] .

Time to reversal was found to be much longer than anticipated, which was similar to other studies in which median time to reversal ranged from 4.1 to 5.9 months [1, 2]. In contrast, however, in a recent systematic review, in which the results by Den Dulk and Gessler were (strikingly) not included, the average time between ileostomy creation and closure was found to be 10.8 weeks [12]. Our study does not confirm the results of this review. One of the reasons for the delay in stoma closure is that stoma closure is considered an elective, low-priority operation that has to compete with more complex and urgent operations. To get around this particular problem, some advocate that setting a date for stoma closure at discharge helps early closure of ileostomies [10].

In our study, 26 % of the temporary ileostomies were not reversed, and if they were, reversal was not without morbidity and even mortality. This means that one out of four patients received a permanent stoma, while in a small percentage of the patients, another stoma had to be constructed because of anastomotic leakage or because of faecal incontinence. The complication rate we found after reversal is higher than the data given in the Chow review. This is probably due to the long follow-up, even after closure, in our patient series.

### Strengths and limitations of this study

The strength of this study is the large size of our patient series with intentionally temporary ileostomies with a long follow-up, which gives a good insight into the practice of a large secondary referral and teaching hospital in the Netherlands.

A limitation of this study is its retrospective nature. As such, it has its possible sources of bias. However, the data were collected from the hospital’s surgical complication registration database combined with the prospectively collected dataset of the enterostomal therapists, which warrants an extensive and reliable registry.

Second, our patient group was rather heterogeneous. However, this variety in patient mix was a stipulation to assess possible risk factors. On the other hand, this was a single-centre study with a limited group of patients and surgeons who cared for them. Therefore, we believe the results of this study are valid and may well be applicable to other centres.

## Conclusion

A substantial number of ileostomies that are intended to be temporary will never be reversed. In case of reversal, the morbidity is considerable. The interval between construction and reversal and the length of hospital stay are substantially longer than usually assumed. It is essential that surgeons are aware of these findings and tailor their surgical strategy to the patients and its disease keeping this in mind. Besides a temporary ileostomy, there are two other options: no diversion or a permanent colostomy. Shared decision-making is to be preferred in these situations [21].
